# Systematic review with meta-analysis: the efficacy and safety of stem cell therapy for Crohn’s disease

**DOI:** 10.1186/s13287-017-0570-x

**Published:** 2017-06-06

**Authors:** Yun Qiu, Man-ying Li, Ting Feng, Rui Feng, Ren Mao, Bai-li Chen, Yao He, Zhi-rong Zeng, Sheng-hong Zhang, Min-hu Chen

**Affiliations:** Department of Gastroenterology, The First Affiliated Hospital of Sun Yat-Sen University, 58 Zhongshan II Road, Guangzhou, 510080 People’s Republic of China

**Keywords:** Crohn’s disease, Stem cells, Meta-analysis, Efficacy, Adverse effects

## Abstract

**Background and aims:**

Stem cell therapy (SCT) for the treatment of Crohn’s disease (CD) is still in its infancy, and whether SCT is associated with improved outcomes is unclear. We performed a meta-analysis to evaluate the efficacy and safety of patients receiving SCT.

**Methods:**

Electronic databases were searched for studies that reported the use of stem cells for the treatment of patients with CD. Raw data from included studies were pooled for effect estimates. Subgroup analyses were performed for exploration of heterogeneity regarding all outcomes.

**Results:**

We analyzed 21 studies comprising 514 patients with active CD. A random-effects meta-analysis of studies of SCT as systemic infusion showed 56% (95% confidence interval (CI) 33–76, *n* = 150) of patients achieved clinical response. Similarly, random-effects pooled rates of clinical or endoscopic remission were 46% (95% CI 25–69, *n* = 116) and 15% (95% CI 0–50, *n* = 48), respectively. A random-effects meta-analysis of all perianal CD studies showed that 57% (95% CI 44–69%, *n* = 251) of patients had healed fistula with SCT, with an odds ratio of 3.83 (95% CI 1.06–13.86, *n* = 121, *P =* 0.04) versus control. The pooled rate of clinical recurrence was high at 16% (95% CI 4–34, *n* = 101) with follow-up >12 months. The pooled rates of severe adverse events (SAEs) and SAEs related to SCT were 12% (95% CI 6–23, *n* = 378) and 8% (95% CI 3–18, *n* = 378), respectively. The Egger test suggests no publication bias existed for fistula healing (*P* = 0.36), but did for clinical response (*P* = 0.003).

**Conclusions:**

SCT seems potentially effective and may serve as an alternative treatment for refractory active CD. Toxicity will remain the most significant barrier to systemic SCT in patients with CD.

**Electronic supplementary material:**

The online version of this article (doi:10.1186/s13287-017-0570-x) contains supplementary material, which is available to authorized users.

## Background

Crohn's disease (CD) is a chronic relapsing inflammatory condition of the gastrointestinal tract that can result in lifelong ill health [1]. Immunosuppressive drugs, including biologicals, are the standard of care for CD, but some patients do not respond or lose response to treatment [2, 3]. Therefore, alternative treatments for refractory CD remain an unmet need.

Hematopoietic stem cell transplantation (HSCT) might play a role in some of these treatment-resistant cases [4]. Previous studies have indicated that allogeneic HSCT may reset the immune system at a genetic level [5, 6], and autologous HSCT eliminates aberrant clones by immunoablation and replacement with uncommitted stem cells (SCs), leading to de novo generation of an altered T-cell repertoire [7]. Case reports and series describe long-term treatment-free disease regression with autologous [8–10] and allogeneic [5, 6] HSCT in some [6, 11, 12] but not all patients [8–10] with CD. However, the recent Autologous Stem Cell Transplantation International Crohn Disease (ASTIC) trial [13] failed to demonstrate a statistically significant improvement in sustained disease remission at 1-year of autologous HSCT compared with conventional therapy. These findings raise doubt about HSCT for patients with refractory CD.

Perianal fistulas are very common complications, appearing in 25% of CD patients. Of these, approximately half are complex. The only approved drug that has shown efficacy in a randomized clinical trial is infliximab [14, 15]. Few treatment options exist for drug treatment-refractory patients, and repeated surgical options are associated with a significant risk of permanent stoma. These findings emphasize the need for novel treatment options for treatment-refractory complex perianal fistulas in patients with CD. Initial clinical results [16, 17] suggested mesenchymal stem cells (MSCs) might have therapeutic potential in this setting.

Given the immunoregulatory potential of SCs, multiple studies have been conducted to assess the safety and efficacy of stem cell therapy (SCT) in CD. In this study, we perform a meta-analysis of feasibility and toxicity of SCT for the treatment of CD.

## Methods

The study was performed following the preferred reporting items for systematic reviews and meta-analyses (PRISMA) guidelines [18].

### Literature search

We identified relevant literature (published articles and abstracts) by performing a systematic search of three databases: PubMed, Cochrane Library CENTRAL, and Embase (initial search February 5, 2015; updated October 15, 2016). Keywords used were (all fields): (“inflammatory bowel disease” or “crohn’s disease” or “crohn disease” or “enteritis”) and (“stem cell*” OR “precursor cell*” OR “progenitor cell*” OR “Stromal cell*”), and any appropriate abbreviations. For PubMed, all relevant MeSH terms were used. The final queries were validated by manual review and matching results.

The conference proceeding abstracts for annual meetings of European (European Crohn’s and Colitis Organisation congress) and American (Digestive Disease Week) Congresses were searched between 2002 and 2016.

### Study selection

We finally performed a manual selection of studies which satisfied the following criteria: (a) observational (prospective or post hoc analysis of prospectively obtained cohort) or interventional design (randomized or non-randomized); (b) established diagnosis of CD by accepted criteria not in clinical or endoscopic remission at study outset; and (c) clear definition of efficacy and adverse events (AEs). Studies with exclusively pediatric patients (<15 years), a diagnosis of ulcerative colitis, indeterminate colitis, or an unclear diagnosis of CD and case reports were also excluded.

### Data extraction and quality assessment

Eligible articles were reviewed in a blind manner by two different investigators (YQ and MYL), and the results of the primary research studies were abstracted onto specially designed data extraction forms. Disagreement in data extraction was resolved by consensus with MHC.

Assessment of the quality of randomized controlled trials (RCTs) and observational studies was performed using the Cochrane risk of bias tool [19] and Newcastle Ottawa Quality Assessment Scale (NOS) [20], respectively. For the NOS, studies scoring >7 (of 9) were considered high quality.

### Outcomes assessed

The primary endpoint was clinical efficacy (e.g., clinical response or remission, fistula healing as defined by the primary study) of SCs for the treatment of patients with CD. Secondary outcomes were safety (e.g., AEs, severe AEs (SAEs)) and other measures of disease activity (endoscopic remission, clinical recurrence) of SCT for CD.

### Data synthesis and analysis

We calculated incidence estimates with the variance-stabilizing double arcsine transformation [21] because the inverse variance weight in fixed-effects meta-analyses is suboptimum when dealing with binary data with low incidences. Additionally, the transformed incidences are weighted very slightly towards 50%, and studies with incidences of zero can thus be included in the analysis. We used the Wilson method [22] to calculate 95% confidence intervals (CIs) around these estimates because the asymptotic method produces intervals which can extend below zero. Heterogeneity due to study variation was assessed using the χ^2^ test [23, 24], with a *P* value of <0.10 being considered as statistically significant.

### Publication bias

Funnel-plot asymmetry as proposed by Egger et al. [25] was used to investigate the possibility of publication bias.

The meta-analysis was performed using the metaprop command of the meta package in R (version 3.2.0) [26] and Stata (version 12.1) with the commands metareg (for metaregression). All statistical tests were two-sided, and statistical significance was defined as a *P* value <0.05.

### Outcomes

**Fig. 1:**
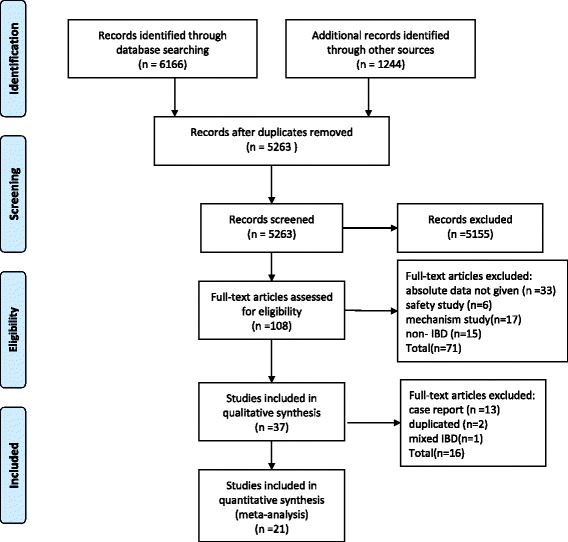
Study selection

The initial search strategy yielded 4828 abstracts for review, of which 108 were selected for detailed review. Seventy-one studies were excluded for being non-human or safety studies or studies with exclusively pediatric patients, or for having a diagnosis of UC or indeterminate colitis or mixed irritable bowel syndrome for which the CD data could not be separated. Sixteen studies were further excluded because they were case studies or duplicate studies (Fig. 1; Additional file 1: Table S1).

The main characteristics of the included 20 studies [9, 10, 13, 16, 27–42] are detailed in Tables 1 and 2. The included two Park et al. studies [32, 37] and two Cho et al. studies [33, 38] were not duplicated. Among the included 20 studies, three [9, 10, 13] were about the systemic infusion of hematopoietic stem cells, seven [27, 29–31, 36, 39, 40] the systemic infusion of bone marrow-derived MSCs (BMSCs), and ten [16, 28, 32–35, 37, 38, 41, 42] the local injection of MSCs; the numbers of transplanted SCs ranged from 10^7^ to 1.5 × 10^10^. All studies defined clinical or endoscopic response with the Crohn’s Disease Activity Index (CDAI) and CD Endoscopic Index of Severity (CDEIS). All 15 observational studies were considered as high quality (scoring >7) using the NOS and the 2/6 RCTs [13, 27, 28, 37, 39, 42] had low risk of bias. Detailed quality assessments are provided in Additional file 2: Figure S1, Additional file 1: Table S1 and Additional file 3: Table S2.

**Table 1:** Characteristics of included studies evaluating the efficacy and safety of stem cell therapy for patients with Crohn’s disease

Study	Number of subjects	Sex (M:F)	Age (years): mean (SD)	Duration (years): mean (SD)	Prior medications, n(%)			Bowel surgery	Concomitant medications				CDAI: mean (SD)	Design	Location
					IM	Biological	Steroid		IM	Biological	Steroid	5-ASA			
Burt et al. 2010 [9]	24	12:12	27	10	Y	Y	Y	71% (17/24)	N	N	Y	-	235	Phase I/IIa open label, single arm	USA
Cassinotti et al. 2012 [43]	10	7:3	-	-	-		-	-	-	-	-	-	-	Phase I/IIa open label, single arm	Italy
Hasselblatt et al. 2012 [10]	12	8:4	36.5 (range 24–50)	12.7 (range 2–24)	Y	Y	Y	8	Y	Y	Y	Y	285 ± 79	Phase I/IIa open label, single arm	Germany
Hawkey et al. 2015 [13]	23	11:13	Median 34.1	14.9 (IQR,9.9-16.9)	-	-	N	2 (IQR,0.5–3.5)	N	N	N	-	326 (range 251–414)	RCT, multicenter	European
Onken et al. 2006 [27]	10	-	-	-	-	-	-	-	-	-	-	-	-	Phase II, open label, double arm, randomized	USA
Duijvestein et al. 2010 [29]	10	2:8	Median 32.5	3 ~ 12	Y	Y	Y	4	Y	N	Y	Y	Median 299.5 (range 255–442)	Phase I, open label, single arm	Netherlands
Forbes et al. 2014 [36]	16	6:10	21–55	-	-	Y	-	3	Y	N	Y	-	>250	Phase II, open label, single arm multicenter	Australia
Lazebnik et al. 2010 [30]	50 (11 CD)	-	-	-	-	-	-	-	N	N	Y	-	261.5 ± 18.2 (range 206–298)	Open label, double arm	Russia
Dhere et al. 2016 [40]	12	6:6	18–52	-	Y	Y	N	-	N	N	N	-	>220	Phase I	USA
Garcia-Olmo et al. 2005 [16]	5	3:2	35.1 ± 2.4	-	Y	Y	Y	Y	N	N	N	N	-	Phase I, open label, single arm	Spain
Garcia-Olmo et al. 2009 [28]	49 (14 CD)	10:14	42.64 ± 10.93	-	6 (25)	N	-	Fistula:17 (71)	-	-	-	-	-	Phase II, open label, double arm, randomized	Spain
Ciccocioppo et al. 2011 [31]	12	8:4	Median 32	-	Y	Y	Y	Y	Y	N	Y	Y	294 ± 49	Open label, single arm	Italy
Park et al. 2012 [32]	11	6:5	24.6	-	-	5	-	-	-	-	-	-	-	Phase I/IIa open label, single arm	Korea
de la Portilla et al. 2013 [34]	24	11:13	36 ± 9	-	-	-	-	-	Y	N	Y	Y	-	Phase I/IIa open label, single arm	Spain
Cho et al. 2013 [33]	10	4:6	26.5 ± 6	-	-	-	-	-	-	-	-	-	-	Phase I, open label, single arm	Korea
Lee et al. 2013 [35]	33	22:11	26.7 ± 5.6	54.6 ± 40.1 months	-	-	-	-	41 (95.3)	-	-	-	-	Phase II, open label, single arm	Korea
Park et al. 2014 [37]	6	-	-	-	-	-	-	-	-	-	-	-	-	Multicenter, randomized phase I/IIa	Korea
Cho et al. 2015 [38]	41	28:13	26.2 ± 5.5	-	-	-	-	-	-	-	-	-	-	f/u of phase II	Korea
Molendijk et al. 2015 [39]	21	-	-	-	-	-	-	-	-	-	-	-	-	Double-blind RCT	Netherlands
Lightner et al. 2016 [41]	7	3:4	31	4.5	Y	Y	Y	Y	-	-	-	-	-	Phase I	USA
Panés et al. 2016 [42]	212	60:47	39 ± 13	12.1 ± 10	89 (83%)	83 (78%)	6/103 (5%)	-	44 (41%)	37 (35%)	6/103 (5%)	-	88.7 ± 48.8	Phase III double-blind RCT multicenter	European, Israel

*Abbreviations*: *CD* Crohn’s disease, *CDAI* Crohn Disease Activity Index, *N* no, *Y* yes, *5-ASA* 5-Aminosalicylic acid, *IM* immunomodulators

**Table 2:** Characteristics of included studies evaluating the efficacy and safety of stem cell therapy for patients with Crohn’s disease

Study	Procedure		Infusion	Number of cells	Outcome	Criteria: clinical remission	MRI	Remission/response/closure (number of patients, time of evaluation)	SAEs	Infections	Recurrence (number of patients, time of evaluation)
	Autologous	Source									
Burt et al. 2010 [9]	Y	HSCs	S	2. 10E6/kg	CDAI, CSI	CDAI <150	-	Clinical: 24 (6–12 m)	1 not related	11	9% (12 m)
						CSI <12					37% (24 m)
						Asymptomatic with no IM					43% (36 m)
Cassinotti et al. 2012 [43]	Y	HSCs	S	Unknown	CDAI	-	-	Clinical: 10 (3 m)	None	-	20% (12 m)
								Endoscopic: 5 (3 m)			50% (24 m)
Hasselblatt et al. 2012 [10]	Y	HSCs	S	Unknown	CDAI	CDAI <150	-	Clinical: 5 (6 m)	4 related	-	7 (37.2 m)
								Endoscopic: 5 (6 m)	Fever, renal failure		
Hawkey et al. 2015 [13]	Y	HSCs	S	3 ~ 8.10E6/kg	CDAI	Sustained disease remission:	-	2/23 versus 1/22 (12 m)	76 versus 38 related	9 SAEs in 5 patients undergoing HSCT:	-
(ASTIC)						1) CDAI <150 for >3 m		10 versus 2 (1 year)		3 EBV reactivation, 2 CMV reactivation,	
						2) No active treatment in the last 3 m				1 herpes zoster, 1 BK virus, 1 intestinal adenovirus, 1 VZV	
						3) No mucosal erosion or ulceration in GI tract				8 neutropenic sepsis	
Onken et al. 2006 [27]	N	Prochymal™	S	Unknown	CDAI		-	Clinical: 9/3 (14 d)	1 unrelated	None	-
Duijvestein et al. 2010 [29]	Y	BMSCs	S	2 × 1/2.10E6/kg	CDAI, CDEIS	Remission: CDAI <150	-	Clinical: 3/0 (6 w)	None	None	-
						Response: a drop in CDAI >70		Endoscopic: 2/0 (6 w)			
Lazebnik et al. 2010 [30]	N	BMSCs	S	150–200.10E8	CDAI, RCAI, Mayo, and Gebs scales	-	-	Clinical: 11/0 (4–8 m, CD)	-	None	-
								39/0 (4–8 m, UC)			
Forbes et al. 2014 [36]	N	BMSCs	S	4 × 2.10E6/kg	CDAI, CDEIS	Remission: CDAI <150	-	Clinical: 12/8 (42 d)	1 unrelated	1 gastroenteritis	-
						Endoscopic improvement: CDEIS <3 or a decrease by >5		Endoscopic: 7/0 (42 d)			
Dhere et al. 2016 [40]	Y	BMSCs	S	2/5/10.10E6/kg	CDAI	Response: CDAI + CRP	-	Clinical: 5/- (2 w)	7	1 acute appendicitis	-
									2 related	1 *C. difficile* colitis	
Garcia-Olmo et al. 2005 [16]	Y	ASCs	L	Unknown	Fistula closure	-	N	8 fistulas in 4: 2/6 (8 w)	None	None	-
Garcia-Olmo et al. 2009 [28]	Y	ASCs + Fg:25 (7 CD)	L	20/60.10E6	Fistula closure	-	N	ASCs + Fg: -/1 (7 CD, 8 w)	None	Perianal sepsis: ASC group (*n* = 3, 12%) versus Fg alone (*n* = 9, 36%) (*P* = 0.04).	Group B: 3 (12 m)
		Fg:24 (7 CD)						Fg: 2/5 (7 CD, 8 w)			No data for CD
Ciccocioppo et al. 2011 [31]	Y	BM-MSCs	L	50.10E6	Complete closure	CDAI + PDAI	Y	3/7 (12 m)	None	None	None (12 m)
Park et al.2012 [32]	Y	ASCs + Fg	L	1 ~ 4.10E7	Fistula healing	-	N	-/9 (8 w)	-	-	-
de la Portilla et al. 2013 [34]	N	ASCs	L	20/60.10E6	Fistula closure	-	Y	23 fistulas in 22: 5/18 (6 m)	2	Infections and infestations 9/7	-
										Anal abscess 5/4	
										Anal fistula infection 2/2	
Cho et al. 2013 [33]	Y	ASCs	L	10/20/40.10E6	Fistula healing	-	N	1/3 (2 m)	None	3 enterocolitis	3 (8 m)
Lee et al. 2013 [35]	Y	ASCs	L	30/60.10E6	Fistula healing	-	N	5/27 (2 m)	None	No ASC-related AEs	3 (12 m)
										1 grade 4 peritonitis caused by CD	
Park et al. 2014 [37]	N	ASCs	L	10/30.10E6	Complete closure	-	N	-/1 (8 w)	-	-	-
								-/3 (8 m)			
Cho et al. 2015 [38]	Y	ASCs	L	30/60.10E6	Fistula healing	-	N	28/35 (12 m)	None	None	3/26 (12 m)
								27/36 (24 m)			4/24 (24 m)
Molendijk et al. 2015 [39]	Y	BMSCs (*n* = 15)	L	10/30/90.10E6	Fistula healing	-	Y	8 versus 1 (week 6)	None	None	-
		Placebo						7 versus 2(wk 12)			
		(*n* = 6)									
								9 versus 2 (week 24)			
Lightner et al. 2016 [41]	Y	ASCs	L	Unknown	Fistula healing	-	Y	6/6 (6 m)	-	-	-
Panés et al. 2016 [42]	N	ASCs (Cx601)	L	120.10E6	Combined remission	-	Y	53/107 versus 36/105	SAE: 18/103 versus 14/103	Anal abscess (6 versus 9)	-
		Saline							SAE related: 5/103 versus 7/103	Proctalgia (5 versus 9)	

*Abbreviations*: *ASC* adipose-derived MSC, *BMSC* bone marrow-derived MSC, *CD* Crohn’s disease, *CDAI* Crohn Disease Activity Index, *CDEIS* CD Endoscopic Index of Severity, *CSI* Crohn Severity Index, *Fg* fibrin glue, *GI* gastrointestinal, *HSC* hematopoietic stem cell, *L* local, *m* month, *N* no, *S* systemic, *SAE* serious adverse event, *w* week, *Y* yes, *RCAI* Rahmilevich Clinical Activity Index, *PDAI* perianal disease activity index, *IM* immunomodulators, *CRP* C-reactive protein, *UC* ulcerative colits, *VZV* varicella-zoster virus

### Efficacy of SCT as a systemic infusion for CD

#### Clinical response

**Fig. 2:**
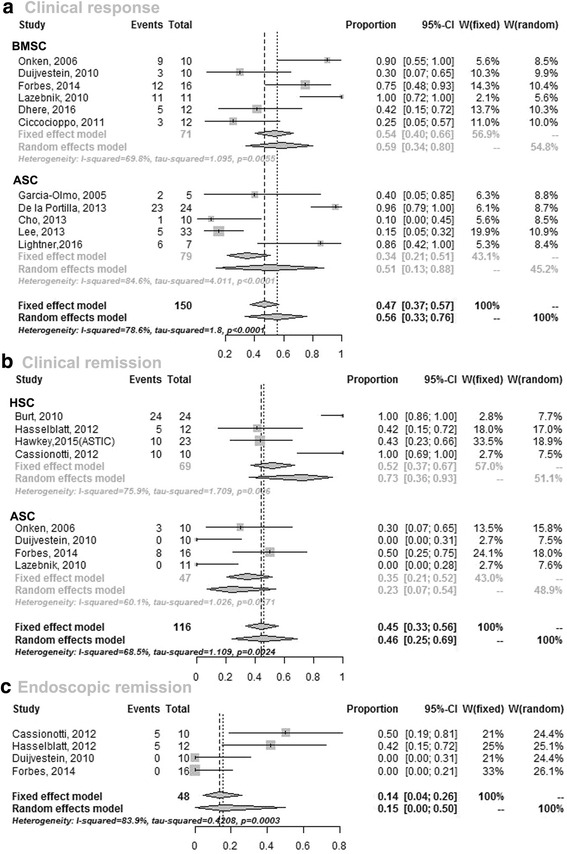
Estimated incidence of **a** clinical response, **b** clinical remission, and **c** endoscopic remission among CD patients who received SCT. *ASC* adipose mesenchymal stem cell, *BMSC* bone marrow-derived mesenchymal stem cell

Eleven studies [16, 27, 29–31, 33–36, 40, 41] reported raw data on clinical response of patients with SCT. Rates of clinical response ranged from 10 to 100%. The random-effects pooled rate of clinical response was 56% (95% CI 33–76, *n* = 150); heterogeneity was pronounced (χ^2^ = 1.88, *P* < 0.001; *I* ^2^ = 78.6%; Fig. 2a). Subgroup analyses were performed to assess whether the source of the stem cells, i.e., HSCT or MSCT, and the method of SC infusion had substantial effects on the efficacy of SCs as well as decreased the high heterogeneity.

Six studies [27, 29–31, 36, 40] reported raw data on the outcome of patients with bone marrow-derived MSC (BMSC) therapy. The pooled rate of clinical response was 59% (95% CI 34–80, *n* = 79) compared to 51% (95% CI 13–88, five studies [16, 33–35, 41], *n* = 71) for patients who had ASC infusion (Table 3).

**Table 3:** Summary of subgroup analysis of stem cells for the treatment of patients with CD

	Clinical response					Clinical remission					Fistula closure				
Items	n	Heterogeneity		ES	95% CI	n	Heterogeneity		ES	95% CI	n	Heterogeneity		ES	95% CI
		*I* ^*2*^	*P*				*I* ^*2*^	*P*				*I* ^*2*^	*P*		
Overall	11	78.6%	<0.001	0.56	[0.33–0.76]	8	68.5%	0.002	0.46	[0.25–0.69]	12	59.9%	0.003	0.57	[0.44–0.69]
Source															
HSC	0	-	-	-	-	4	75.9%	0.006	0.73	[0.36–0.93]	2	80.8%	0.022	0.29	[0.03–0.85]
BMSC	6	69.8%	0.005	0.59	[0.34–0.80]	4	60.1%	0.057	0.23	[0.07–0.54]	2	0.0%	0.740	0.60	[0.44–0.75]
ASC	5	84.6%	<0.001	0.51	[0.13–0.88]	0	-	-	-	-	9	65.3%	0.003	0.62	[0.42–0.79]
Source															
Autologous	7	49.6%	0.064	0.31	[0.17–0.49]	5	76.4%	0.002	0.61	[0.25–0.88]	9	59.5%	0.011	0.62	[0.44–0.77]
Allogeneic	4	26.7%	0.251	0.86	[0.71–0.94]	3	56%	0.103	0.32	[0.11–0.62]	4	30.2%	0.231	0.47	[0.33–0.61]
Routine															
Systemic	5	67.7%	0.001	0.66	[0.39–0.86]	8	68.5%	0.002	0.46	[0.25–0.69]	2	80.8%	0.023	0.29	[0.03–0.85]
Local	6	81.0%	<0.001	0.45	[0.16–0.79]	0	-	-	-	-	11	57.9%	0.002	0.60	[0.47–0.72]

*Abbreviations*: *ASC* adipose-derived mesenchymal stem cell, *BMSC* bone marrow-derived mesenchymal stem cell, *HSC* hematopoietic stem cell

Seven studies [16, 29, 31, 33, 35, 40, 41] reported results in patients with autologous SCT. The pooled rate of clinical response was 31% (95% CI 17–49, *n* = 89) compared to 86% (95% CI 71–94, four studies [27, 30, 34, 36], *n* = 61) for patients who underwent allogeneic SCT (Table 3).

The pooled rate of clinical response was 66% (95% CI 39–86, five studies [27, 29, 30, 36, 40], *n* = 59) in patients who received systemic SCT compared to 45% (95% CI 16–79, six studies [16, 31, 33–35, 41], *n* = 91) for patients with local SCT (Table 3).

#### Clinical remission

Eight studies [9, 10, 13, 27, 29, 30, 36, 43] reported raw data on clinical remission (mainly defined as a CDAI <150; Tables 1 and 2) of patients with SCT. A random-effects meta-analysis of all studies of SCT as systemic infusion showed that 46% (95% CI 25–69, *n* = 116) of patients achieved clinical remission after infusion of SCs (Fig. 2b). Heterogeneity was modest (χ^2^ = 1.11, *P* = 0.002; *I* ^2^ = 68.5%).

Four studies [9, 10, 13, 43] reported raw data on the outcome of patients with HSCT. The pooled rate of clinical remission was 73% (95% CI 36–93, *n* = 69) in patients who received HSCT compared to 23% (95% CI 7–54, four studies [27, 29, 30, 36], *n* = 47) of patients who received MSCT (Table 3).

#### Endoscopic remission

Four studies [10, 29, 36, 43] reported raw data on the endoscopic remission of patients with SCT. Rates of endoscopic remission ranged from 0–50% (Fig. 2c). The random-effects pooled rate of endoscopic remission was 15% (95% CI 0–50, *n* = 48); heterogeneity was pronounced (χ^2^ = 0.42, *P* < 0.001; *I* ^2^ = 83.9%).

#### Efficacy of SCT for perianal CD (local therapy)

**Fig. 3:**
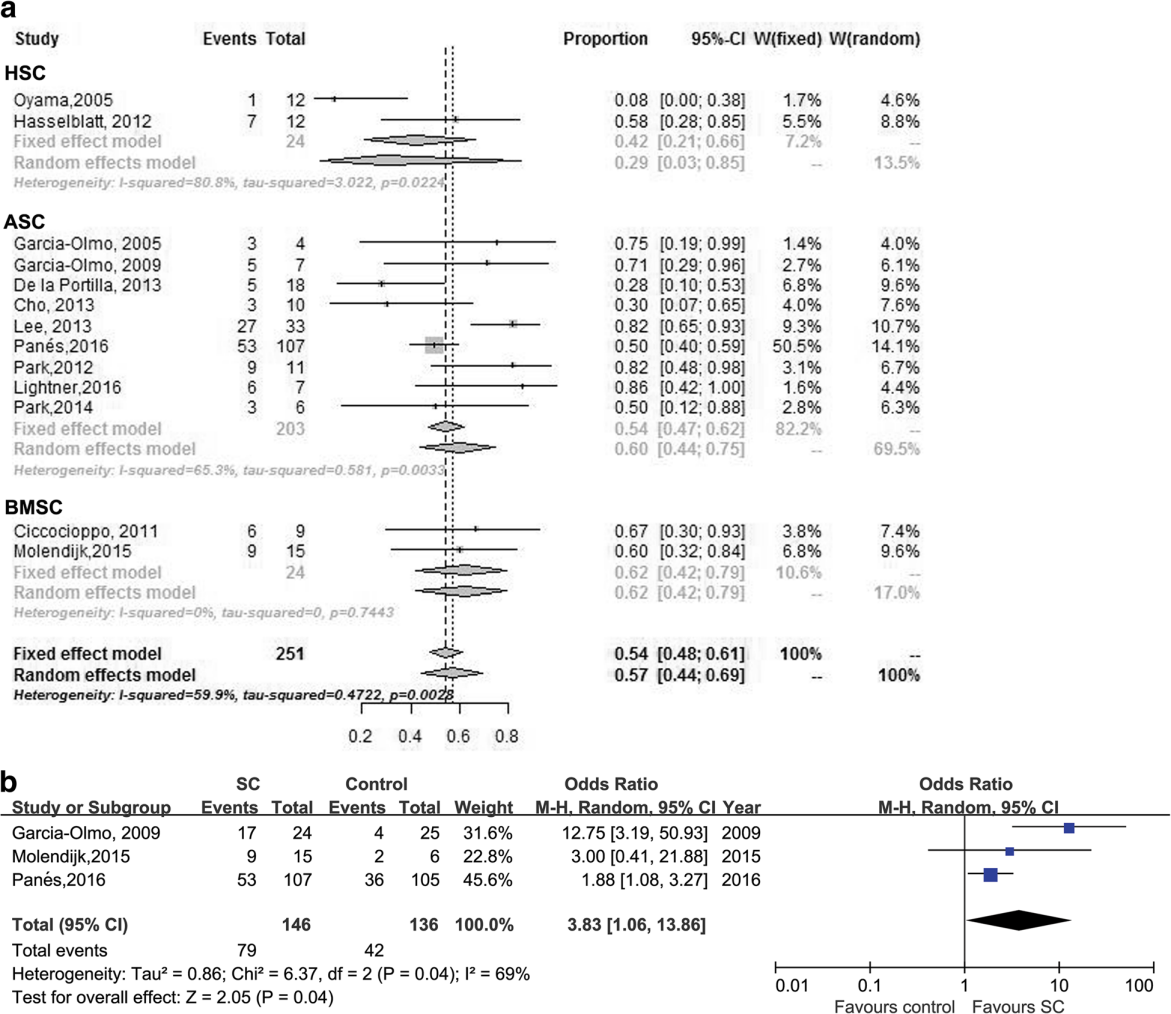
Forest plot of **a** studies evaluating healing of perianal fistulas (perianal CD) with SCT. **b** Comparison of SCT versus control. *ASC* adipose mesenchymal stem cell, *BMSC* bone marrow-derived mesenchymal stem cell, *HSCT* hematopoietic stem cell

Thirteen studies [8, 10, 16, 28, 31–35, 37, 39, 41, 42] reported raw data on the efficacy of SCT for perianal CD of patients with SC injection. A random-effects meta-analysis of all perianal CD studies showed that 57% (95% CI 44%–69%, *n* = 251) of patients had healed fistula after local MSC injection (Fig. 3a) with mild heterogeneity (χ^2^ = 0.47, *P* = 0.003; *I* ^2^ = 59.9%).

Nine studies [16, 28, 32–35, 37, 41, 42] reported raw data on the outcome of patients who received ASC injection. The random-effects pooled rate of fistula healing was 60% (95% CI 44–75, *n* = 203) compared to 29% (95% CI 3–85, two studies [8, 10], *n* = 24) for patients who received HSCT and 62% (95% CI 42–79, two studies [31, 39], *n* = 24) for patients who received BMSC (Table 3).

Nine studies [8, 10, 16, 31–33, 35, 39, 41] reported results in patients with autologous SCT with a pooled rate of fistula healing of 62% (95% CI 44–77, *n* = 113) compared to 47% (95% CI 33–61, four studies [28, 34, 37, 42], *n* = 138) for patients who underwent allogeneic SCT (Table 3). No heterogeneity existed between the studies.

The pooled rate of fistula healing was 29% (95% CI 3–85, two studies [8, 10], *n* = 24) for patients who received systemic SCT compared to 60% (95% CI 47–72, 11 studies [16, 28, 31–35, 37, 39, 41, 42], *n* = 227) for patients who received local SCT (Table 3).

#### Comparison of SC versus control

Among the 13 studies, five [27, 28, 37, 39, 42] were double-armed; both Onken et al. [27] and Park et al. [37] compared two different doses of SCs. Thus, three studies [28, 39, 42] were pooled, yielding an odds ratio (OR) of 3.83 (95% CI 1.06–13.86, *n* = 121, *P =* 0.04) for achieving fistula healing in patients who received SCT versus control (Fig. 3b). Heterogeneity was modest (χ^2^ = 6.37, *P* = 0.04; *I* ^2^ = 69%).

#### Recurrence

**Fig. 4:**
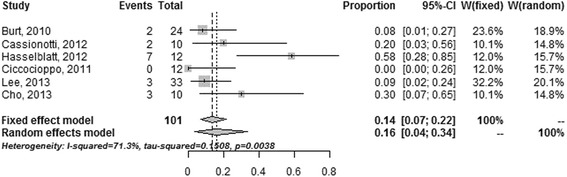
Estimated incidence of clinical recurrence among CD patients who received SCT

Six studies [9, 10, 31, 33, 35, 43] reported raw data on recurrence for patients with SCT. Rates of recurrence ranged from 0 to 58% with a follow-up of >12 months (Fig. 4). The random-effects pooled rate of recurrence was 16% (95% CI 4–34, *n* = 101); heterogeneity was pronounced (χ^2^ = 0.15, *P* = 0.004; *I* ^2^ = 71.3%).

Three studies [9, 10, 43] reported raw data on the outcome of patients with HSCT. The pooled rate of clinical remission was 25% (95% CI 6–64, *n* = 46) for patients who received HSCT compared to 17% (95% CI 5–45, four studies [33, 35], *n* = 43) for patients who received ASCs and 4% (95% CI 0–40, four studies [31], *n* = 12) for patients who received BMSCs.

#### Safety profile

**Fig. 5:**
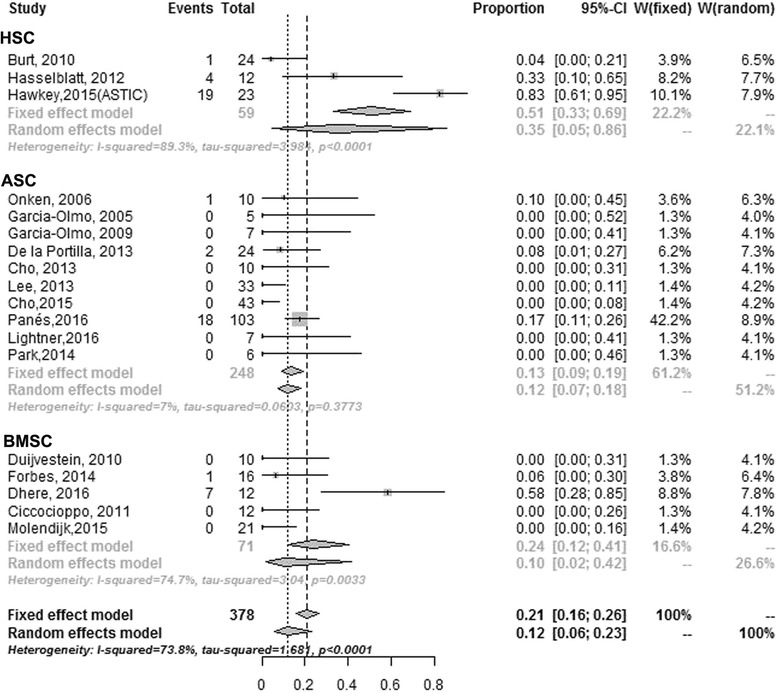
Estimated incidence of SAEs among CD patients who received SCT

Overall, the included studies demonstrate that administration of SCs could lead to minor adverse reactions like perianal sepsis; however, serious adverse reactions leading to hospitalization are less common and perhaps related to underlying CD activity (Table 2). Eighteen [9, 10, 13, 16, 17, 27, 29, 31, 33–42] of 20 studies reported raw data on severe adverse events (SAEs) of patients with SCT. Rates of SAE ranged from 0–83%. The random-effects pooled rate of SAEs was 12% (95% CI 6–23, *n* = 378); heterogeneity was modest (χ^2^ = 1.68, *P* < 0.001; *I* ^2^ = 73.8%; Fig. 5). The random-effects pooled rate of SAEs related to SCT was 8% (95% CI 3–18, *n* = 378); heterogeneity was modest (*P* < 0.001; *I* ^2^ = 74.2.6%).

#### Publication bias and heterogeneity

**Fig. 6:**
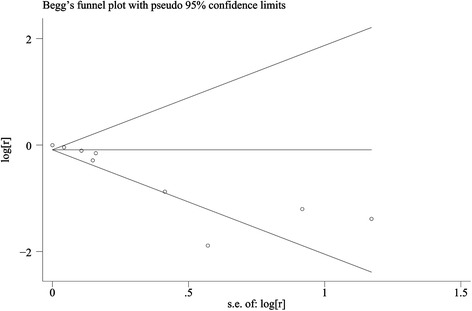
Begg’s funnel plot for publication bias for evaluating clinical response. *S.e.* standard error

A funnel plot generated for the primary outcome suggested no publication bias (Fig. 6), as did an Egger test for fistula healing (t = −0.97, *P* = 0.36), but not for clinical response (t = −4.91, *P* = 0.003).

## Discussion

Our meta-analysis suggests that SCT has good therapeutic potential with relatively low risk of AEs for patients with CD, particularly for those who have perianal disease and are treated with local therapy.

MSCs have regenerative and immunomodulatory properties which lead to reduction of inflammation and healing of affected intestinal tissue. A previous meta-analysis showed that 40.5% (95% CI 7.5–78.5) of patients achieved remission after systemic infusion of MSCs [44] compared to 23% (95% CI 7–54) in our study. Autologous and allogeneic MSC administration are both being evaluated; their unique cell surface HLA characteristics allow for therapy with unrelated donor cells without increasing the risk of rejection by the host. According to our study, the pooled rate of clinical remission was 32% (95% CI 11–62) for patients who underwent allogeneic MSCT compared to 61% (95% CI 25–88) for patients who underwent autologous SCT (Table 3). However, patients who underwent allogeneic MSCT had a relatively higher pooled rate of clinical response compared with patients who underwent autologous SCT (86 versus 31%). Whether allogeneic MSCT has a superior therapeutic efficacy for CD warrants further study.

When considering HSCT, the question remains whether its risk to benefit ratio justifies it. Observations of case reports published to date suggest that sustained clinical remission with HSCT is initially likely to result from lymph ablation by drugs used in the conditioning regimen; altered immune reconstitution may be a later effect. Temporarily disrupting the immunological memory and ceasing the chronic inflammatory burden using non-ablative HSCT has been shown to be effective in non-controlled study designs [9]. However, the recent ASTIC trial [13] failed to demonstrate a statistically significant improvement in sustained disease remission at 1-year of HSCT. According to our study, the pooled rate of clinical remission was 73% (95% CI 36–93) for patients who underwent autologous HSCT, which was higher than 23% (95% CI 7–54) for patients who received ASC. Based on these trial findings, further study of HSCT in patients with refractory CD may be warranted.

When considering fistulizing CD, the existing pharmacological treatments for complex perianal fistulas have low efficacy in inducing fistula healing (antibiotics 21–48%; thiopurines 20–40%; anti-TNFs 23% complete responders) [45]. In our study, the random-effects meta-analysis of all perianal CD studies showed that 57% (95% CI 44–69) of patients had healed fistula after local MSC injection, which is superior to all the above-mentioned treatments. The pooled OR for achieving fistula healing in patients who received SC injection versus control was 3.83 (95% CI 1.06–13.86, *P =* 0.04).

Whether SCT is associated with improved long-term outcomes for CD patients is unclear. Burt et al. [9] reported that the percentage of clinical relapse-free survival after autologous nonmyeloablative HSCT was 91% at 1 year, 57% at 3 years, and 19% at 5 years. The percentage of patients in remission, steroid-free, or medication-free at any post-transplantation evaluation interval more than 5 years after HSCT remained at or greater than 70, 80, and 60%, respectively. Ciccocioppo et al. [31] reported the efficacy of MSCs for refractory complex CD fistulae declined over time and fistula relapse-free survival was 50% at 2 years and 37% at 4 years. Cho et al. [38] reported that 83.3% (20/24) of patients who received MSCs maintained complete fistula closure at 2 years. In the present meta-analysis, the pooled rate of clinical recurrence was 16% (95% CI 4–34) with a follow-up >12 months. Studies assessing the long-term safety and efficacy of systemic MSC therapy for luminal CD are lacking. Further studies that investigate the impact of periodic MSC administration on long-term outcomes are necessary to establish SCT as maintenance therapy.

The safety of SCT remains a major concern that must be addressed before it can be officially approved for use for CD. During the first 12-month follow-up, viral infections were the most commonly observed complications of autologous HSCT [46]. This is further confirmed by the ASTIC trial [13], with most importantly proven or presumed infections associated with the pancytopenia induced by the conditioning regimen. In contrast, Kniazev et al. [47] revealed no differences in the development of acute post-transfusion reactions, infectious complications, exacerbations of chronic inflammatory diseases, severe infectious complications, malignant transformation, and fatal cases in patients who received MSC therapy during 5-year follow-up. In our analysis, the pooled rate of SAEs and SAEs related to SCT were 12% (95% CI 6–23) and 8% (95% CI 3–18), respectively. When subgrouped by source of SCs, patients who received HSCs had a pooled rate of SAEs of 35% (95% CI 5–86), which is higher than with either BMSC (10%, 95% CI 2–42) or ASC (12%, 95% CI 7–18) infusion. MSCT does not require preparatory regimens involving high-dose chemotherapy and/or radiation; HSCT is thus associated with less procedure-related mortality. Overall, autologous HSCT for patients with refractory CD is feasible, but extraordinary supportive measures need to be implemented.

This meta-analysis also has limitations. First, assessment of the methodological quality determined that there were deficiencies in all the studies evaluated. Although only six RCTs [13, 17, 27, 37, 39, 42] met the inclusion criteria, the majority of studies included are phase I/IIa clinical trials. Second, clinical response exhibited statistical heterogeneity, which likely reflects the variability of definitions for clinical response, study design, sources of SCs, and time to clinical or endoscopic assessment. We used a random effects model to conservatively account for the clinical and statistical heterogeneity in the pooled studies. We also performed subgroup analyses to examine differences in the overall effect estimate.

## Conclusions

So far, not enough studies have so far been performed to have a clear view about the use of SCT for CD; up to now the findings are encouraging but not conclusive. SCT seems potentially effective for refractory CD and has high efficacy in inducing fistula healing. Based on subgroup analysis, systemic allogeneic BMSC transfusion had relatively higher rates of inducing clinical response, while autologous ASC local injection had relatively higher rates of inducing fistula closure. Studies are needed to standardize MSC injection in often complex fistulas with multiple tracts to determine optimal dosing and source. Systemic infusion of SCs is not yet ready for the clinic and faces multiple challenges. Toxicity will remain the most significant barrier to HSCT in patients with CD.

## Additional files

Additional file 1: Table S1. Characteristics of excluded studies. (DOCX 35 kb)

Additional file 2: Figure S1. Risk of bias within studies assessed by Cochrane risk of bias assessment tool. (TIF 1067 kb)

Additional file 3: Table S2. Assessment of quality of observational studies using Newcastle Ottawa Quality Assessment Scale (NOS) (DOCX 14 kb)

## Abbreviations

AE: adverse event
ASC: Adipose mesenchymal stem cell
ASTIC: Autologous Stem Cell Transplantation International Crohn Disease
BMSC: Bone marrow-derived MSC
CD: Crohn’s disease
CDAI: Crohn’s Disease Activity Index
CI: Confidence interval
HSCT: Hematopoietic stem cell transplantation
MSC: Mesenchymal stem cell
NOS: Newcastle Ottawa Quality Assessment Scale
OR: Odds ratio
RCT: Randomized controlled trial
SAE: Severe adverse event
SC: Stem cell
SCT: Stem cell therapy
TNF: Tumor necrosis factor-α
