# Supplementary material for: Systematic review with meta-analysis: the efficacy and safety of stem cell therapy for Crohn’s disease
Source: Stem Cell Res Ther. 2017 Jun 6;8:136. doi: 10.1186/s13287-017-0570-x (PMC5460506; doi:10.1186/s13287-017-0570-x)
Supplement: Supplementary file 1 — Characteristics of excluded studies. (DOCX 35 kb) [file 13287_2017_570_MOESM1_ESM.docx]

**TableS 1**. Characters of included studies evaluating the effecacy and safety of stem cell therapy for patients with Crohn's disease.

| **Study** | **design** | **Location** | **Infusion** | **Outcome** | **MRI** | **Participants** | **Procedure** | | **Remission (no. of pts, time of evaluation)** | | | **SAEs** | | **Recurrence (no. of pts, time of evaluation)** | |  |
| --- | --- | --- | --- | --- | --- | --- | --- | --- | --- | --- | --- | --- | --- | --- | --- | --- |
| Lopez-cubero, 1998[^1^](#_ENREF_1) | Case Report | USA | S | Physician | N | 6 (5 evaluable) | Allogeneic HSCs | | | Clinical:4 (4.5-15.3 yr) | | 1SAEs related septicemia | | 1 (1.5 yr) | | |
| Kreisel, 2003[^2^](#_ENREF_2) | Case Report | Germany | S | CDAI | N | 1 | Autologous HSCs | | | Clinical:1 (9 m) Endoscopic:1 (9 m) | | None | | none | | |
| Craig, 2003[^3^](#_ENREF_3) | NA | USA | S | CDAI | N | 6 | Autologous HSCs | | Clinical:6 (range 3-18 m) | | | NA | | NA | |  |
|  |  |  |  |  |  |  |  | | Endoscopic:6/0 (6 m) | | |  | |  | |  |
|  |  |  |  |  |  |  |  | | Closure:6 | | |  | |  | |  |
| Oyama,2005^[4](#_ENREF_4)^ | phase 1 | USA | S | CDAI | N | 12 | Autologous HSCs | | Clinical:11 (12 m) | | | None | | 1 (15 m) | |  |
| Cassinotti, 2007^31^ | Case Report |  | S | CDAI | N | 4 | Autologous HSCT+ | | Clinical:4/4 (3 m) | | | None | | 1(16.5m) | |  |
|  |  |  |  |  |  |  | unselected PBSCs | | Endoscopic:2/3 (3 m) Closure:4(16.5m) | | |  | |  | |  |
| Craig, 2008[^5^](#_ENREF_5) | Open label, cohort, single arm | USA | S | CDAI,CSI | N | 21 | Autologous HSCs | | clinical:18 | | | NA | | 9 | |  |
| Hommes, 2011[^6^](#_ENREF_6) | Case Report | Netherlands | S | CDAI | N | 3 | Autologous HSCs | | Clinical:3 (5-6 yr) | | | None | | 1 (2 yr) | |  |
| Kountouras,2011^[7](#_ENREF_7)^ | Case Report | Greece | S | CDAI | N | 1 | Autologous HSCs | | Clinical:1 (31 m) | | | None | | Clinical:0 (31 m) | |  |
| Nishimoto,2013^[8](#_ENREF_8)^ | Case Report | Japan | S | CDAI | N | 1 | | Allogeneic HSCs | | | Clinical:1 (20 m) | | None | None |  |  |
| Ruiz,2015^[9](#_ENREF_9)^ | Case Report | Brazil | S | CDAI | N | 1 | | Autologous HSCs | | | Clinical:1 (12 m) Endoscopic:1 (6 m) | | None | 0(12 m) |  |  |
| Jauregui, 2016[^10^](#_ENREF_10) | Case Report | Spain | S | CDAI | N | 1 | | Autologous HSCs | | | Clinical:1/1(4 yr) | | None | 0(4 yr) |  |  |
| Dinesen,2009^[11](#_ENREF_11)^ | Case Report |  | S | CDAI | Y | 1 | | Allogeneic BMSCs | | | Clinical:1/0 (14 w) | | None | NA |  |  |
| Liang, 2012[^12^](#_ENREF_12) | NA | China | S | CDAI, CAI | N | 7 (4CD/3UC) | | Allogeneic BMSCs/ | | | Clinical:7/3 (3 m) | | None | 2 (6-7 m) |  |  |
|  |  |  |  |  |  |  | | UC-MSCs | | | Endoscopic:3/0 (3-5 m) | |  |  |  |  |
| Arturo,2014^[13](#_ENREF_13)^ | Case Report | Colombia | S | CDAI | N | 1 | | Autologous BMSCs | | | Clinical: NA/1 (3 m) Clinical: NA/1 (12 m) | | NA | 0(12m) |  |  |
| Garcia-Olmo, 2003[^14^](#_ENREF_14) | Case report | Spain | L | Fistula closure | N | 1 | | Autologous ASCs | | | NA/1(3m) | | None | None |  |  |
| Garcia-Olmo,2009^[15](#_ENREF_15)^ | Case report | Spain | L | Fistula closure | N | 9 | | Allogeneic ASCs | | | NA/4(8w) | | None | None |  |  |

**Abbreviations**: PBSCs, peripheral blood stem cells; CD, Crohn's disease; ASCs, Adipose-derived MSCs; HSCs, hematopoietic stem cells; w, wk; m, month; yr, year; SAEs, serious adverse events; Fg, Fibrin glue; -, not available; CDAI, Crohn Disease Activity Index; CSI, Crohn Severity Index; L, local; S, systemic; Y, yes; N, no.

1. Lopez-Cubero SO, Sullivan KM, McDonald GB. Course of Crohn's disease after allogeneic marrow transplantation. Gastroenterology 1998;114:433-40.

2. Kreisel W, Potthoff K, Bertz H, et al. Complete remission of Crohn's disease after high-dose cyclophosphamide and autologous stem cell transplantation. Bone Marrow Transplant 2003;32:337-40.

3. Craig R, Oyama Y, Traynor A, et al. Bone marrow ablation and autologous hematopoietic stem cell transplantation (HSCT) for severe Crohn's disease (CD). Gastroenterology 2003;124:A520.

4. Oyama Y, Craig RM, Traynor AE, et al. Autologous hematopoietic stem cell transplantation in patients with refractory Crohn's disease. Gastroenterology 2005;128:552-63.

5. Craig RM, Burt RK. 87 Clinical Course Following Autologous, Nonmyeloablative, Stem Cell Transplantation in Patients with Refractory Crohn's Disease, 2001-2007. Gastroenterology 2008;134:A-14.

6. Hommes DW, Duijvestein M, Zelinkova Z, et al. Long-term follow-up of autologous hematopoietic stem cell transplantation for severe refractory Crohn's disease. J Crohns Colitis 2011;5:543-9.

7. Kountouras J, Sakellari I, Tsarouchas G, et al. Autologous haematopoietic stem cell transplantation in a patient with refractory Crohn's disease. Journal of Crohn's and Colitis 2011;5:275-276.

8. Nishimoto M, Nakamae H, Watanabe K, et al. Successful Treatment of Both Acute Leukemia and Active Crohn's Disease After Allogeneic Hematopoietic Stem Cell Transplantation Using Reduced-Intensity Conditioning With Fludarabine and Busulfan: A Case Report. Transplantation Proceedings 2013;45:2854-2857.

9. Ruiz MA, Kaiser Junior RL, Gouvêa Faria MA, et al. Remission of refractory Crohn's disease after autologous hematopoietic stem cell transplantation. Revista Brasileira de Hematologia e Hemoterapia 2015;37:136-139.

10. Jauregui-Amezaga A, Rovira M, Lopez A, et al. Long-lasting Remission Induced by Syngeneic Haematopoietic Stem Cell Transplantation in a Patient with Refractory Crohn's Disease. J Crohns Colitis 2016;10:1122-4.

11. Dinesen LC, Wang A, Vianello F, et al. W1148 Mesenchymal Stem Cells Administered via Novel Selective Mesenteric Artery Cannulation for the Treatment of Severe Refractory Crohn'sDisease. Gastroenterology 2009;136:A-664.

12. Liang J, Zhang H, Wang D, et al. Allogeneic mesenchymal stem cell transplantation in seven patients with refractory inflammatory bowel disease. Gut 2012;61:468-9.

13. Arturo J, Perez C, Larios L, et al. Immunomodulation and induction of remission in crohn's disease with autologous expanded mesenchymal stem cells. case report. Cytotherapy 2014;16:S101.

14. Garcia-Olmo D, Garcia-Arranz M, Garcia LG, et al. Autologous stem cell transplantation for treatment of rectovaginal fistula in perianal Crohn's disease: a new cell-based therapy. Int J Colorectal Dis 2003;18:451-4.

15. Garcia-Olmo D, Herreros D, Pascual M, et al. Treatment of enterocutaneous fistula in Crohn's Disease with adipose-derived stem cells: a comparison of protocols with and without cell expansion. Int J Colorectal Dis 2009;24:27-30.
