# Supplementary figures and images for: Systematic review with meta-analysis: the efficacy and safety of stem cell therapy for Crohn’s disease
Source: Stem Cell Res Ther. 2017 Jun 6;8:136. doi: 10.1186/s13287-017-0570-x (PMC5460506; doi:10.1186/s13287-017-0570-x)

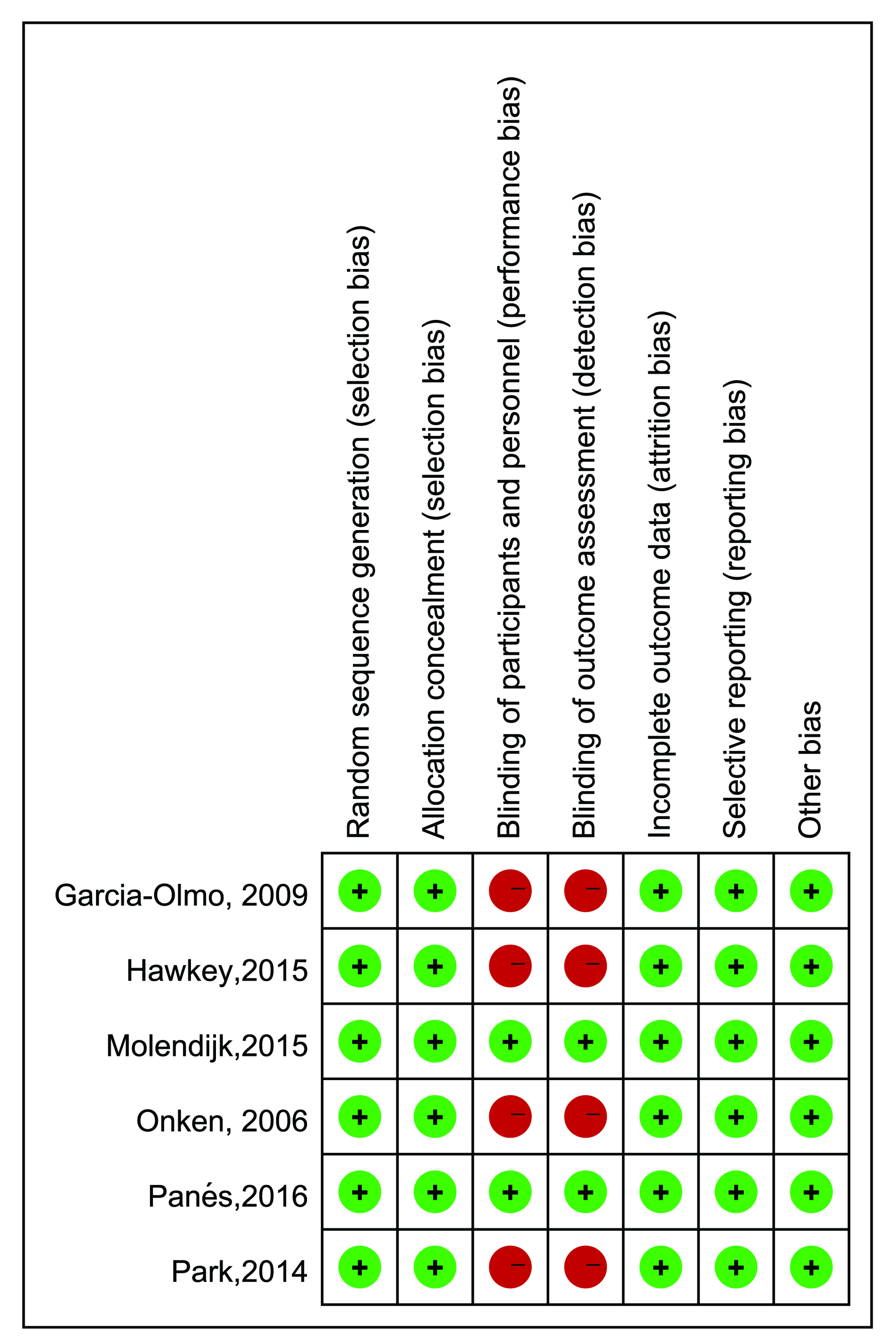

Supplement: Supplementary file 2 — Risk of bias within studies assessed by Cochrane risk of bias assessment tool. (TIF 1067 kb) [file 13287_2017_570_MOESM2_ESM.tif]
