# Supplementary material for: Systematic review with meta-analysis: the efficacy and safety of stem cell therapy for Crohn’s disease
Source: Stem Cell Res Ther. 2017 Jun 6;8:136. doi: 10.1186/s13287-017-0570-x (PMC5460506; doi:10.1186/s13287-017-0570-x)
Supplement: Supplementary file 3 — Assessment of quality of observational studies using Newcastle Ottawa Quality Assessment Scale (NOS) (DOCX 14 kb) [file 13287_2017_570_MOESM3_ESM.docx]

**Table S2.** Assessment of quality of observational studies using Newcastle Ottawa Quality Assessment Scale (NOS)

| **Study** | **Selection** | **Comparability** | **Outcome/ exposure** | **Total score** |
| --- | --- | --- | --- | --- |
|  |  |  |  | **(out of 9)** |
| Garcia-Olmo, 2005 | 3 | 1 | 3 | 7 |
| Oyama,2005 | 3 | 1 | 3 | 7 |
| Craig, 2008 | 3 | 1 | 3 | 7 |
| Burt, 2010 | 3 | 1 | 3 | 7 |
| Duijvestein, 2010 | 3 | 1 | 3 | 7 |
| Lazebnik, 2010 | 3 | 2 | 3 | 8 |
| Ciccocioppo, 2011 | 3 | 2 | 3 | 8 |
| Hasselblatt, 2012 | 3 | 1 | 3 | 7 |
| Park,2012 | 3 | 1 | 3 | 7 |
| Cho, 2013 | 3 | 1 | 3 | 7 |
| De la Portilla, 2013 | 3 | 1 | 3 | 7 |
| Lee, 2013 | 3 | 1 | 3 | 7 |
| Forbes, 2014 | 4 | 2 | 3 | 9 |
| Cho,2015 | 3 | 2 | 3 | 8 |
| Dhere, 2016 | 3 | 1 | 3 | 7 |
| Lightner,2016 | 3 | 2 | 3 | 8 |
